# Effect of Fluoroscopic-Guided Corticosteroid Injection in Patients With Sacroiliac Joint Dysfunction

**DOI:** 10.7759/cureus.36406

**Published:** 2023-03-20

**Authors:** Anurag Patel, Dileep Kumar, Shailendra Singh, Ravindra Mohan, Sudhir Mishra, Anil K Gupta, Ganesh Yadav

**Affiliations:** 1 Orthopaedics, Maa Vindhyawasini Autonomous State Medical College & Associated Divisional District Hospital, Mirzapur, IND; 2 Physical Medicine and Rehabilitation, King George’s Medical University, Lucknow, IND; 3 Orthopaedic Surgery, King George’s Medical University, Lucknow, IND

**Keywords:** sacroiliac joint, intra-articular injection, pain management, dysfunction, corticosteroid

## Abstract

Background

Sacroiliac joint dysfunction is a major cause of axial low back pain which can masquerade as pain from lumbar disc diseases. Treatment of axial back pain arising due to sacroiliac joint dysfunction remains a challenge. This study was conducted to evaluate the long and short-term effects of intra-articular corticosteroid injection in the relief of pain and disability caused by sacroiliac joint dysfunction.

Methodology

A total of 83 patients with sacroiliac joint dysfunction were included in this prospective randomized control study. Patients were randomized into two groups by a computer-generated randomization table. These two groups were treated with fluoroscopy-guided corticosteroid and local anesthetic injection (group A) and distilled water and local anesthetic injection (group B). Pre and post-intervention assessment of all patients was done based on the Numeric Pain Rating Scale (NPRS) for pain and Oswestry Disability Index (ODI) for disability. The outcome measures of the study were the NPRS and ODI assessed at the initial visit one (pre-injection), two weeks post-injection (visit 2), and four weeks post-injection (visit 3).

Results

Demographic data were comparable in both groups. There was no significant difference in pre-injection NPRS and ODI values in both groups. The changes in NPRS and ODI values were significant from pre-injection to two weeks to four weeks. Group A patients performed better in terms of a decrease in the perception of pain and a decrease in the perception of disability compared to group B patients in the second and fourth weeks of follow-up.

Conclusions

Fluoroscopy-guided corticosteroid injection is an effective measure for reducing pain and disability in patients with sacroiliac joint dysfunction.

## Introduction

A significant contributing factor to persistent axial low back pain is the sacroiliac joint [1]. With age, its frequency rises [2]. It is estimated that sacroiliac joint dysfunctions cause 15-30% of cases of low back pain [3]. The dorsal rami of the sacral nerves are primarily responsible for the joint’s innervation. The sacroiliac joint can cause intra-articular, extra-articular, or unidentified pain. Fractures, ligamentous injuries, and myofascial pain are extra-articular causes of sacroiliac joint pain. Intra-articular causes of sacroiliac joint pain include infection, arthritis, spondyloarthropathies, and malignancies [4].

Pain in the lower back and/or legs can occasionally result from sacroiliac joint dysfunction. Because they can feel very similar, it can be challenging to distinguish between radiating leg pain from a lumbar disc herniation (sciatica) and sacroiliac joint dysfunction. According to the International Association for the Study of Pain (IASP), sacroiliac joint pain is described as being exclusive to the sacroiliac joint area, reproducible through stress and provocation testing of the joint, and consistently reduced by 50% by elective local anesthetic injection of the joint [5]. Because the patient continues to experience pain, functional impairment, and related psychiatric co-morbidity as a result of conservative treatment of sacroiliac joint pain with analgesics, this lowers their quality of life.

The diagnosis often starts with the collection of medical history, which includes details of the present pain and symptoms. Medical history also includes details of a person’s diet, sleeping, and physical activity routines, as well as any current or previous injuries that may have contributed to the development of sacroiliac joint pain. Gaenslen’s test, Patrick’s test, Yeoman’s test, side-lying iliac compression test, midline sacral thrust, and pressure application to the sacral sulcus while in the prone position are the provocation tests for the diagnosis of sacroiliac joint dysfunction [6,7].

Treatments for sacroiliac joint dysfunction (sacroiliac joint pain) usually concentrate on reducing discomfort and regaining the joint’s range of motion. The majority of sacroiliac joint pain can be treated without surgery.

Initial therapies for sacroiliac joint discomfort frequently involve a brief moment of rest. Resting for more than a few days is not advised because it can exacerbate stiffness, increase pain, and lead to general deconditioning. Ice helps reduce swelling and ease pain and discomfort when administered to the pelvis and low back. For mild-to-moderate pain management, over-the-counter analgesics (such as acetaminophen) and non-steroidal anti-inflammatory drugs (NSAIDs, such as ibuprofen or naproxen) can be prescribed. No single treatment works for everyone. Typically, multiple non-invasive procedures must be used to effectively relieve pain. Additionally finding medications that target particular symptoms can require a trial and error.

Literature supports the use of intra-articular sacroiliac joint injections and radiofrequency thermoneurolysis for both short- and long-term relief. Sacroiliac joint dysfunction is one condition for which fluoroscopy-guided corticosteroid injections are used; therefore, evaluating the advantages of sacroiliac joint injection in patients with sacroiliac joint dysfunction/pain will expand the scope of currently available treatments for chronic low back pain and pave the way for new areas of study in the management of chronic low back pain. This prospective study was conducted to determine the efficacy of fluoroscopy-guided sacroiliac joint corticosteroid injection in the management of patients with chronic axial low back pain secondary to sacroiliac joint dysfunction/pain who presented to our center [8].

## Materials and methods

This study evaluates the effect of fluoroscopy-guided corticosteroid injection in patients with sacroiliac joint dysfunction. In total, 83 patients with sacroiliac joint dysfunction who were age and sex-matched were assessed for eligibility. Of these, eight patients did not meet the inclusion criteria, and three patients refused to participate in the study. Hence, 72 patients were randomized into two groups by a computer-generated randomization table. Both groups were treated with fluoroscopy-guided corticosteroid and local anesthetic injection (group A) and distilled water and local anesthetic injection (group B).

This prospective randomized control trial was conducted at King George’s Medical University, Lucknow, after obtaining ethical clearance from the institutional ethical board (97th ECM II B Thesis/P62) and informed consent from all patients. The study group comprised 72 subjects. Patients in the age group of 18-60 years with pain located over the sacroiliac joint principally below the L5 vertebra and satisfying the IASP diagnosis criteria of sacroiliac joint dysfunction were included in the study. Patients with spinal and pelvic deformities or disorders of the hip joint and those with allergic reactions to lidocaine or contrast media were excluded from the study. Further, patients with suspected or diagnosed infection, poor general health, skin defects in the injection area, and patients with uncontrolled diabetes were not included in this study to avoid generalized infections.

Simple randomization was done. All patients who fulfilled the inclusion criteria and gave written informed consent for participation in the study were enrolled with concealed allocation method using a computer-generated system. The principal investigator generated the random allocation sequence and enrolled the participants and allowed the intervention. Regarding blinding, only the trial participants were blinded in the study.

In the procedure room, patients were connected to a multi-parameter monitor to obtain baseline vital signs, and periodic cardiorespiratory parameters and venous access were secured. With patients in the prone position, the C-arm fluoroscope was placed in the anteroposterior (AP) position to visualize the L5-S1 disc space at the L5-S1 interspace. The widest space at the most inferior aspect of the sacroiliac joint was identified, and the C-arm was angled 5° in the cephalad direction and 15° in the medial direction until the lines of the anterior and posterior aspects of the joint overlapped. The needle entry point was at the inferior edge (distal 1 cm) of the joint, and confirmation of the needle was done by injecting contrast media. After confirmation, group A was injected with 1.0 mL of triamcinolone acetonide and 1 mL of 2% lignocaine. In group B, 1 mL of distilled water and 1 mL of 2% lignocaine were administered. All patients were promoted to exercise for 20 minutes three times a week for four weeks. The Numeric Pain Rating Scale (NPRS) for pain and Oswestry Disability Index (ODI) for disability were assessed at week zero (pre-intervention, visit one) and week two (visit two) and week four (visit three) (post-intervention) during follow-up. Pre-intervention assessment of all patients was done using the NPRS for pain and ODI for disability [9,10].

## Results

**Figure 1:**
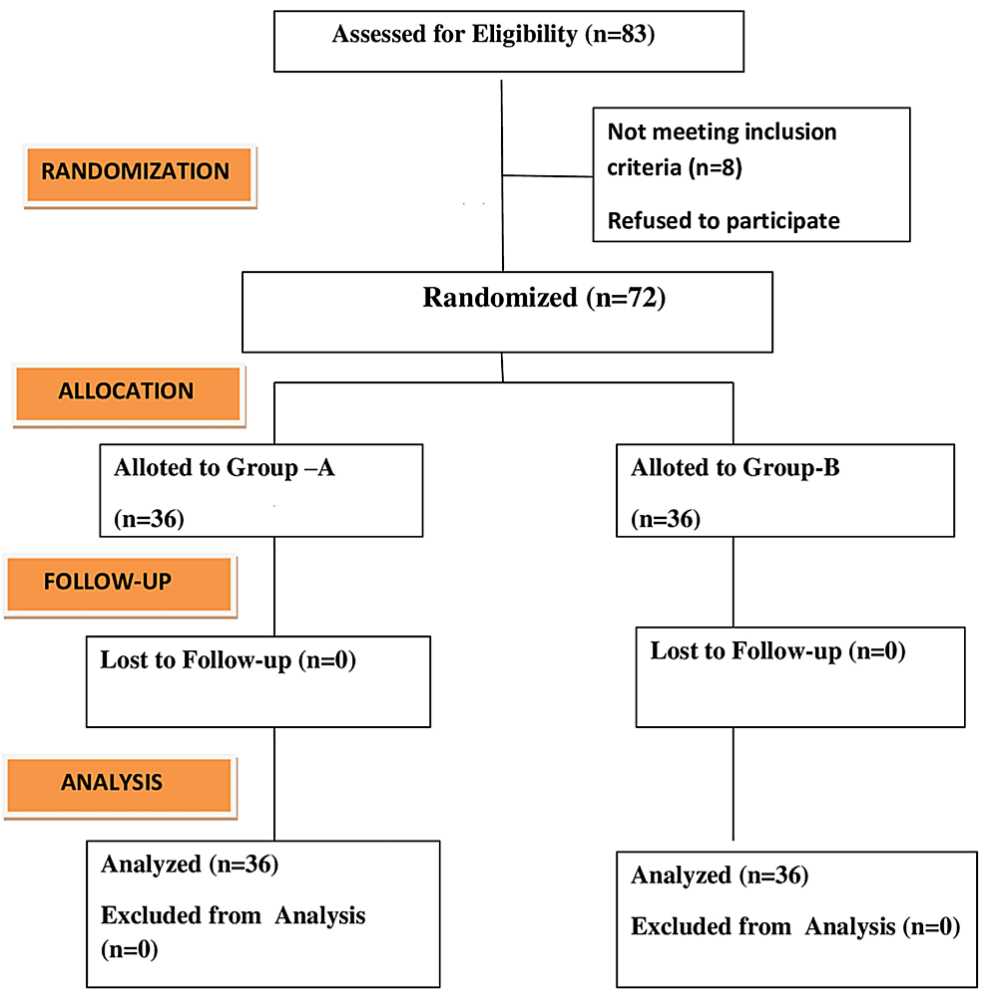
Consort flow diagram.

A total of 83 patients were enrolled in the study but three refused to participate and eight did not meet the inclusion criteria; hence, 72 patients were randomly allocated to two groups of 36 each, as shown in the consort flow diagram (Figure 1).

No significant differences were found in age, gender, site, and group distribution. The outcome measures of the study were NPRS and ODI assessed at the initial visit one (pre-injection), two weeks post-injection (visit two), and four weeks post-injection (visit three) in both groups.

At pre-injection, the mean NPRS in group A was 6.97 ± 0.878 and in group B was 7.28 ± 0.701. At pre-injection, group A mean ODI was 35.94 ± 0.841 and group B mean ODI was 37.08 ± 0.729. No significant difference (p = 0.107) was found between the average pre-injection NPRS and pre-injection ODI of the two groups (Table 1).

**Table 1:** Baseline comparison of the groups based on the NPRS and ODI. SD = standard deviation; SE = standard error; CI = confidence interval; NPRS = Numeric Pain Rating Scale; ODI = Oswestry Disability Index

	Group	N	Mean	SD	SE mean	t-test	P-value
NPRS pre-injection	A	36	6.97	0.878	0.146	-1.632	0.107
	B	36	7.28	0.701	0.117		
ODI pre-injection	A	36	35.94	0.841	0.841	-1.023	0.310
	B	36	37.08	0.729	0.729		

It was observed that estimated marginal mean value of NPRS in group A decreased significantly from the pre-injection value of around 7 to two weeks and four weeks post-injection value in the range of 3 and 4. Hence, with each follow-up, the pain was reduced in patients receiving a sacroiliac joint injection and physical therapy. Physical therapy consisted of a combination of muscle stretches, strengthening exercises, and heat therapy. NPRS values were statistically significantly different between pre-injection and the second and fourth weeks of follow-up, but the value was not significantly different between the second and fourth weeks (Table 2).

**Table 2:** Pair-wise comparisons of the NRPS in group A. *: significant; b: adjustment for multiple comparisons using Bonferroni. SE = standard error; CI = confidence interval; NPRS = Numeric Pain Rating Scale

Mean NPRS at time (I)	Mean NPRS at time (J)	(I-J) Mean difference	SE	P val-e	95% CI for difference^b^	
					Lower bound	Upper bound
Pre-injection (6.97)	Second week (3.14)	3.833^*^	0.220	0.000	3.280	4.387
Pre-injection (6.97)	Fourth week (3.06)	3.917^*^	0.274	0.000	3.227	4.606
Second week (3.14)	Fourth week (3.06)	0.083	0.180	1.000	0.369	0.536

The estimated marginal mean value of NPRS in group B was statistically significantly different between pre-injection and the second and fourth weeks of follow-up, but the value was not significantly different between the second and fourth weeks (Table 3).

**Table 3:** Pair-wise comparison of the NPRS in group B. *: significant; b: adjustment for multiple comparisons using Bonferroni. SE = standard error; CI = confidence interval; NPRS = Numeric Pain Rating Scale

Mean NPRS at time (I)	Mean NPRS at time (J)	(I-J) Mean difference	SE	P-value	95% CI for difference^b^	
					Lower bound	Upper bound
Pre-injection (7.28)	Second week (4.47)	2.806^*^	0.238	0.000	2.206	3.405
Pre-injection (7.28)	Fourth week (5.17)	2.111^*^	0.272	0.000	1.426	2.796
Second week (4.47)	Fourth week (5.17)	0.694	0.284	0.059	-0.020	1.409

The NPRS value decreased in all time frames of follow-up in group A, but in group B, it increased in the fourth week from the second week value. Group A patients showed better performance in terms of decrease in perception of pain than group B patients in both the second and fourth weeks of follow-up (Table 4).

**Table 4:** NPRS final point follow-up in two groups. SD = standard deviation; SE = standard error; CI = confidence interval; NPRS = Numeric Pain Rating Scale

	Group	N	Mean	SD	SE mean	t-test	P-value
NPRS second week	A	36	3.14	1.246	0.208	-4.069	<0.001
	B	36	4.47	1.521	0.254		
NPRS fourth week	A	36	3.06	1.567	0.261	-5.699	<0.001
	B	36	5.17	1.577	0.263		

**Figure 2:**
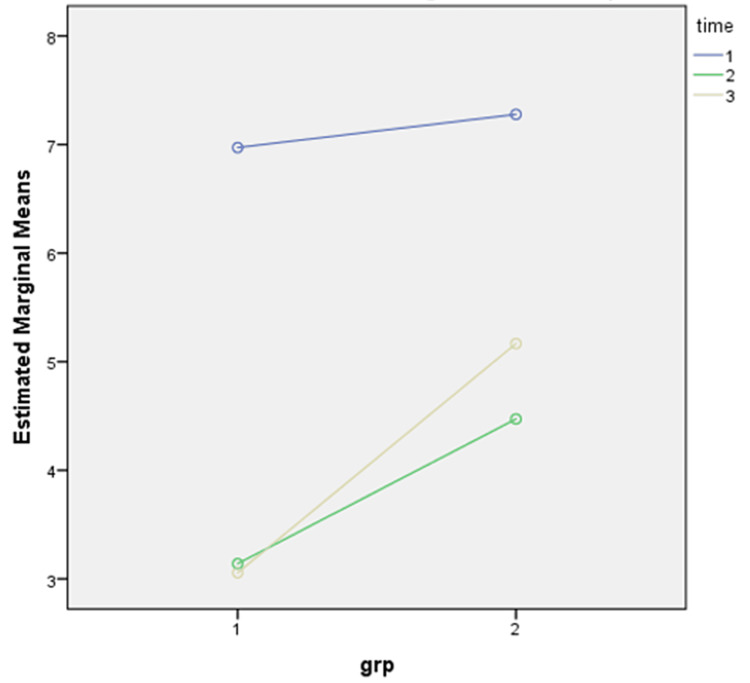
Graph showing the estimated marginal means of NPRS. Group 1 = group A; Group 2 = group B; Time 1 = pre-injection; Time 2 = two weeks post-injection; Time 3 = four weeks post-injection; NPRS = Numeric Pain Rating Scale

The estimated marginal mean value of NPRS at pre-injection ranged between 6 and 7 in both the groups, whereas after two weeks, the estimated marginal mean value of NPRS decreased and ranged between 0 and 3 in group A and between 4 and 5 in group B. After four weeks of follow-up, the estimated marginal mean value of NPRS was between 0 and 3 in group A and between 5 and 6 in group B. This showed that interventions in both groups had beneficial effects after four weeks, but the effect was more beneficial and long-lasting in group A compared to that in group B (Figure 2).

On pair-wise comparison of ODI values in group A, it was found that there was a statistically significant difference from pre-injection to the second to fourth weeks of follow-up, but the value was not significantly different between the second and fourth weeks (Table 5).

**Table 5:** Pair-wise comparison of the ODI in group A. *: significant; b: adjustment for multiple comparisons using Bonferroni. SE = standard error; CI = confidence interval; ODI = Oswestry Disability Index

Mean ODI at time (I)	Mean ODI at time (J)	(I-J) Mean difference	SE	P-value	95% CI for difference^b^	
					Lower bound	Upper bound
Pre-injection (35.94)	Second week (19.58)	16.361^*^	1.121	0.000	13.543	19.179
Pre-injection (35.94)	Fourth week (18.14)	17.806^*^	1.452	0.000	14.155	21.456
Second week (19.58)	Fourth week (18.14)	1.444	0.916	0.371	-0.859	3.747

ODI value in group B was statistically significantly different between all time frames of follow-up, i.e., pre-injection and the second and fourth weeks of follow-up (Table 6).

**Table 6:** Pair-wise comparison of the ODI in group B. *: significant; b: adjustment for multiple comparisons using Bonferroni. SE = standard error; CI = confidence interval; ODI = Oswestry Disability Index

Time (I)	Time (J)	(I-J) Mean difference	SE	P-value	95% CI for difference^b^	
					Lower bound	Upper bound
Pre-injection (37.02)	Second week (24.17)	12.917^*^	1.162	0.000	9.994	15.840
Pre-injection (37.02)	Fourth week (29.06)	8.028^*^	1.294	0.000	4.773	11.283
Second week (24.17)	Fourth week (29.06)	4.889^*^	1.156	0.000	1.982	7.796

Group A patients performed better in terms of decrease in perception of disability than group B patients at both the second and fourth weeks of follow-up. ODI value decreased in all time frames of follow-up in group A, but in group B, it increased in the fourth week from the second week value. This can be explained by the fact that in group B we injected local anaesthetic which has less duration of effects compared to group A in which we injected corticosteroid which shows longer duration of action. Group A patients performed better in terms of decrease in perception of disability than group B patients at both the second and fourth weeks of follow-up (Table 7).

**Table 7:** ODI final point follow-up in two groups. SD = standard deviation; SE = standard error; ODI = Oswestry Disability Index

	Group	N	Mean	SD	SE mean	t-test	P-value
ODI second week	A	36	19.58	6.491	1.082	-2.697	0.009
	B	36	24.17	7.865	1.311		
ODI fourth week	A	36	18.14	8.932	1.489	-5.461	<0.001
	B	36	29.06	8.003	1.334		

**Figure 3:**
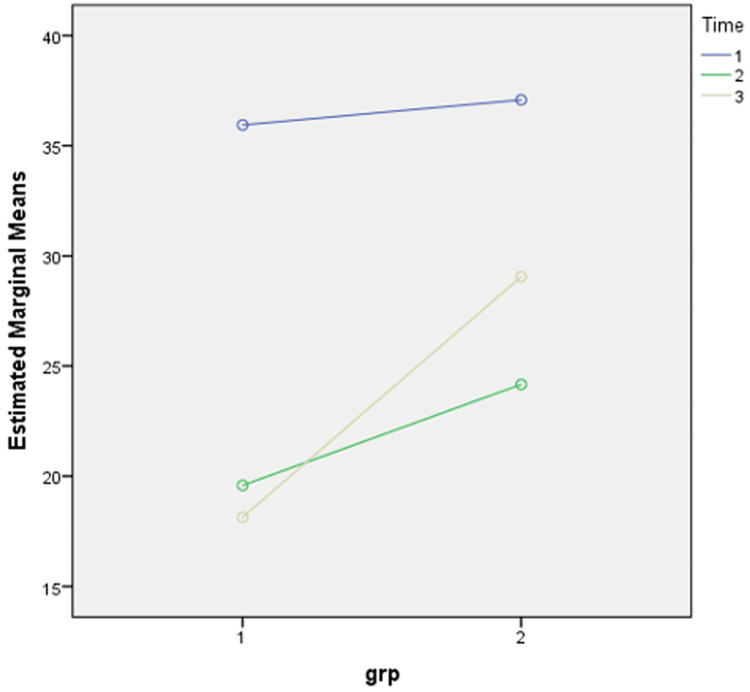
Estimated marginal mean of ODI. Group 1 = group A; Group 2 = group B; Time 1 = pre-injection; Time 2 = two weeks post-injection; Time 3 = four weeks post-injection; ODI = Oswestry Disability Index

The estimated marginal mean value of ODI at pre-injection ranged between 35 and 40 in both groups, whereas two weeks post-injection, the estimated marginal mean value of ODI decreased and ranged between 15 and 20 in group A and between 20 and 25 in group B. After four weeks of follow-up, the estimated marginal mean value of ODI was between 15 and 20 in group A and between 25 and 30 in group B (Figure 3).

This showed that interventions in both groups had beneficial effects after the four-week follow-up period, but the effect was more beneficial and long-lasting in group A compared to that in group B. This can be explained by the fact that local anaesthetic has less duration of action compared to corticosteroid which shows longer duration of action.

## Discussion

The sacroiliac joint is an underrated cause of low back pain, causing at least 15% of low back pain [11]. Longstanding pain arising from sacroiliac joint dysfunction may be accompanied by distressing cognitive behavioral changes, ultimately leading to stress, lack of sleep, mood swings, and depression and affecting the daily and professional activities of patients. The pelvic anatomy is very complex, with the joint space being variable, irregular, and very less in space. The sacroiliac joint is distinctive by virtue of possessing elements of combined synarthrosis and diarthrosis. The main part of the sacroiliac joint is surrounded by a complex capsule and lined with cartilage (diarthrosis). Moreover, it is auricular in shape and opens on the posterior side in particular movements. The sacrum and ilium have an extracapsular, dorsally located articulation (synarthrosis), which is reinforced by the extensive interosseous sacroiliac ligament that provides great internal stability. History and physical examination can be helpful in screening for sacroiliac joint pain, but individual provocative maneuvers have unproven validity. Fluoroscopically guided injections into the joint have been found to be helpful for diagnostic and therapeutic purposes [12,13].

In our study, we found that after two weeks of injection, the mean NPRS was 3.14 v 1.246 in group A (p < 0.001) and 4.47 ± 1.521 in group B (p <0.001). After four weeks of injection, the mean NPRS was 3.06 ± 1.567 in group A (p < 0.001) and 5.171 ± 0.577 in group B (p < 0.001). A previous study found that the mean NPRS was 1.67 ± 0.18 (p < 0.001) after one week and 0.94 ± 0.15 (p < 0.001) after four weeks in their fluoroscopy group [14]. This shows the reduction in NPRS values in group A after two and four weeks compared to that in group B, but in group B NPRS value decreases after two weeks similar to that in group A, and NPRS value increases in group B compared to that in group A. This shows that improvement in pain and disability in group A was significant compared to that in group B even four weeks post-injection.

In our study, we found that after two weeks of injection, the mean ODI was 19.58 ± 6.491 in group A and 24.17 ± 7.865 in group B (p = 0.009), and after four weeks of injection, the mean ODI was 18.14 ± 8.932 in group A and 29.06 ± 8.003 in group B (p < 0.001). A previous study found that the mean ODI was 27.50 ± 0.86 after four weeks (p < 0.001) in the fluoroscopy group [14]. In our study, we also administered paracetamol 500 mg tablet on an SOS basis post-injection and counted the pills. We found that in group A, there was less need for paracetamol compared to group B. This shows that improvement in pain and disability in sacroiliac joint dysfunction or sacroiliac joint pain in group A was significant compared to that in group B.

In our study, we have demonstrated that fluoroscopy-guided corticosteroid injection in patients with sacroiliac joint dysfunction is effective for up to one month of follow-up period compared to the placebo group. This randomized controlled trial reported improvements in each of the two treatment groups over the course of the study, suggesting some degree of efficacy of both treatments. Improvements in both groups were seen when assessed by the NPRS and ODI scores. Our study showed that this intervention, i.e., fluoroscopy-guided sacroiliac joint injection, not only reduces pain but also decreases disability and gives clinicians a proven efficacious short-term treatment for patients with sacroiliac joint dysfunction/pain.

Limitations

Although the experienced clinician may have controlled the location of the needle probe, the free-hand technique used for intra-articular delivery may have affected the results of this study. This study is a single-blinded randomized controlled trial study and could have been a double-blinded randomized controlled study. With respect to the findings of our study, these limitations make it advisable to design future studies that consider low effect sizes and long-term follow-ups, as well as include larger sample sizes.

## Conclusions

There was a significant improvement in low back pain due to sacroiliac joint dysfunction after fluoroscopy-guided corticosteroid injection for the duration of one month, as reflected by the decrease in the NPRS at two weeks and four weeks post-injection follow-ups. Despite limited evidence of long-term effectiveness, corticosteroids have been shown to be more effective in short time periods, as shown by a significant reduction in NPRS and ODI values in group A participants in comparison to that in group B participants.
